# Supplementary material for: Cracking the neural code for word recognition in convolutional neural networks
Source: PLoS Comput Biol. 2024 Sep 6;20(9):e1012430. doi: 10.1371/journal.pcbi.1012430 (PMC11410253; doi:10.1371/journal.pcbi.1012430)
Supplement: S1 Text — Fig A: Discriminating languages with same script. Fig B: Representation space span for letters. Fig C: Examples of units with mixed selectivity. Section A: Connectivity between IT and V4 units. Fig D: Functional connectivity between V4 and IT units (edge position tuning). Fig E: Functional connectivity between V4 and IT units (mid position tuning). Section B: Connectivity between V4 and V2 units. Fig F: Functional connectivity between V2 and V4 units (edge position tuning). Fig G: Functional connectivity between V2 and V4 units (mid position tuning). Section C: Emergence of blank space coding units. Fig H: Visualization of V1 filters. Fig I: Functional connectivity between V1 and V2 layers. Fig J: Activation-maximization of word selective units (negative direction). (DOCX) [file pcbi.1012430.s001.docx]

**Cracking the neural code for word recognition in convolutional neural networks**

Aakash Agrawal, Stanislas Dehaene

*Fig A: Discriminating languages with same script*

*Fig B: Representation space span for letters*

*Fig C: Examples of units with mixed selectivity*

*Section A: Connectivity between IT and V4 units*

*Fig D: Functional connectivity between V4 and IT units (edge position tuning)*

*Fig E: Functional connectivity between V4 and IT units (mid position tuning)*

*Section B: Connectivity between V4 and V2 units*

*Fig F: Functional connectivity between V2 and V4 units (edge position tuning)*

*Fig G: Functional connectivity between V2 and V4 units (mid position tuning)*

*Section C: Emergence of blank space coding units*

*Fig H: Visualization of V1 filters*

*Fig I: Functional connectivity between V1 and V2 layers.*

*Fig J: Activation-maximization of word selective units (negative direction)*


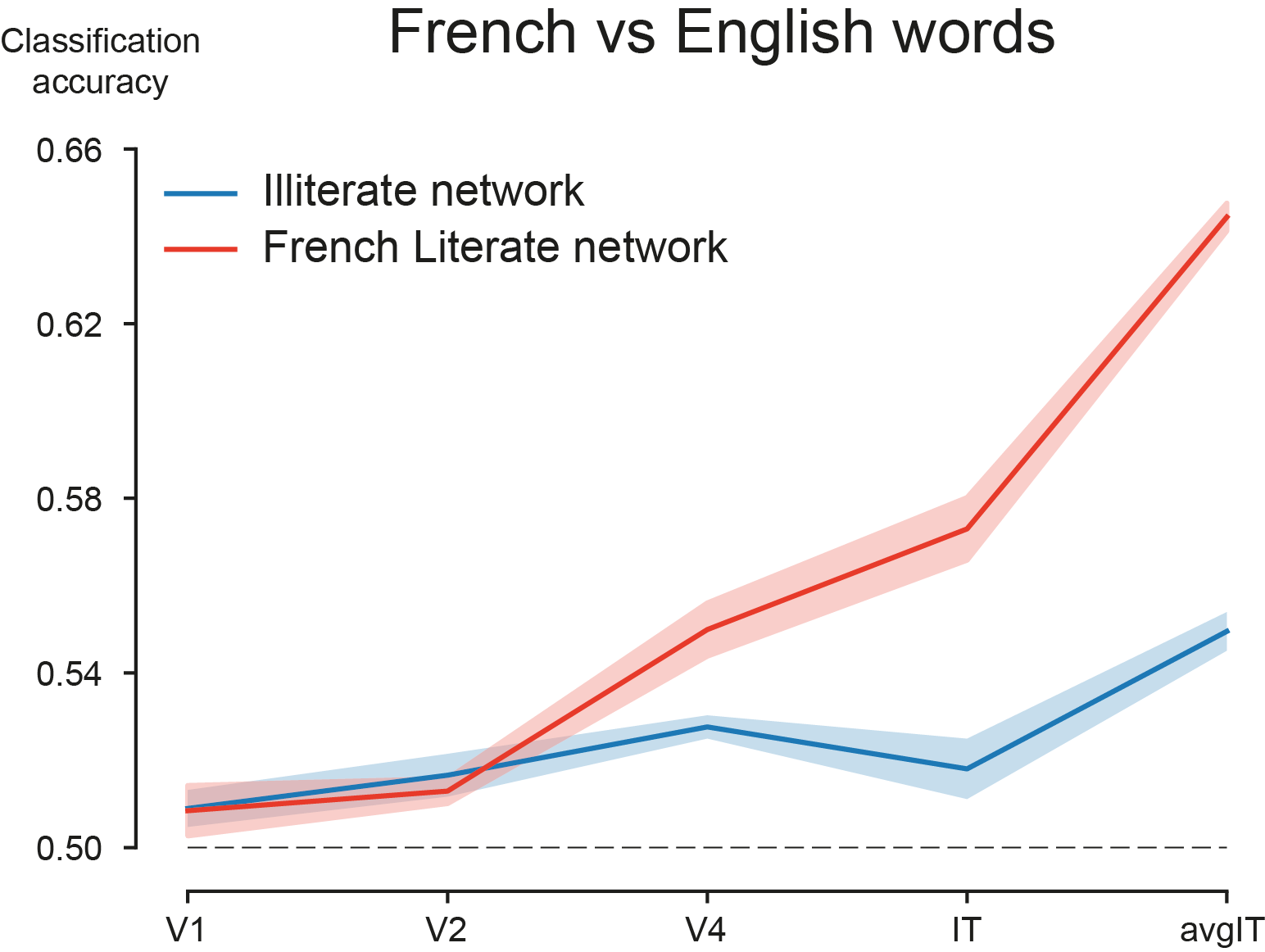


**Fig A: Discriminating languages with same script.** Classification accuracy between English and French words (n = 959 each, excluding 41 common stimuli) across different layers of the French literate (red) and illiterate (blue) network. The word-selective units within each layer were used as features, and 5-fold cross-validation was performed to avoid over-fitting. The shaded error bars indicate standard error of mean across 5 instances of each network.


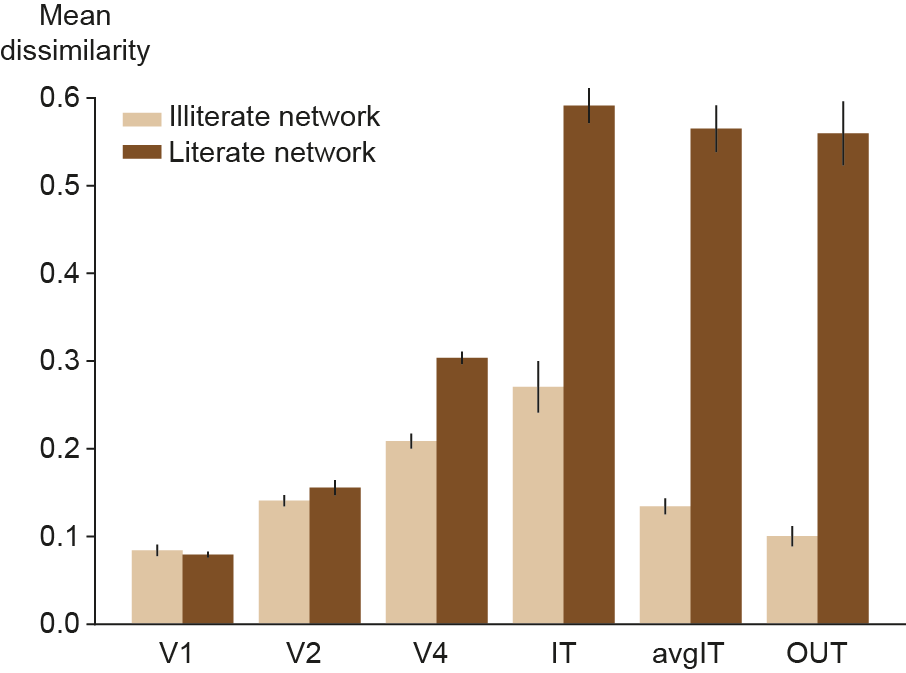


**Fig B: Representation space span for letters.** Mean dissimilarity **(**Same as Fig 1C) but for 26 letters presented at 8 spatial positions along the horizontal axis (n = ^208^C_2_ = 21528) from different layers of French literate (dark) and illiterate (light) networks.


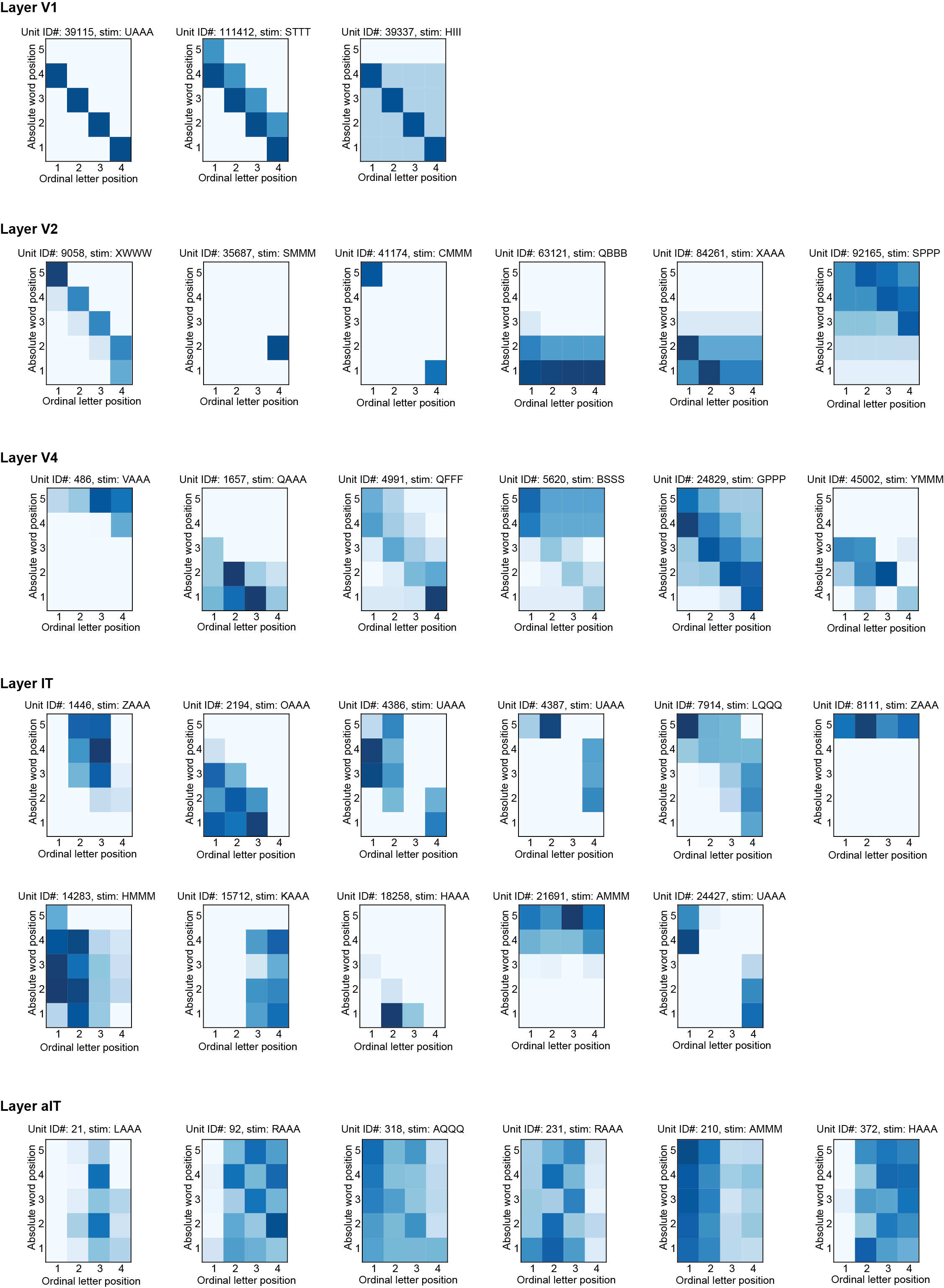


**Fig C: Examples of units with mixed selectivity.** Diversity of response profile across word-selective units from different layers of the literate network. Plots for each word selective unit can be visualised under position_tuning.zip folder at (https://osf.io/926fs)

**Section A: Connectivity between IT and V4 units**


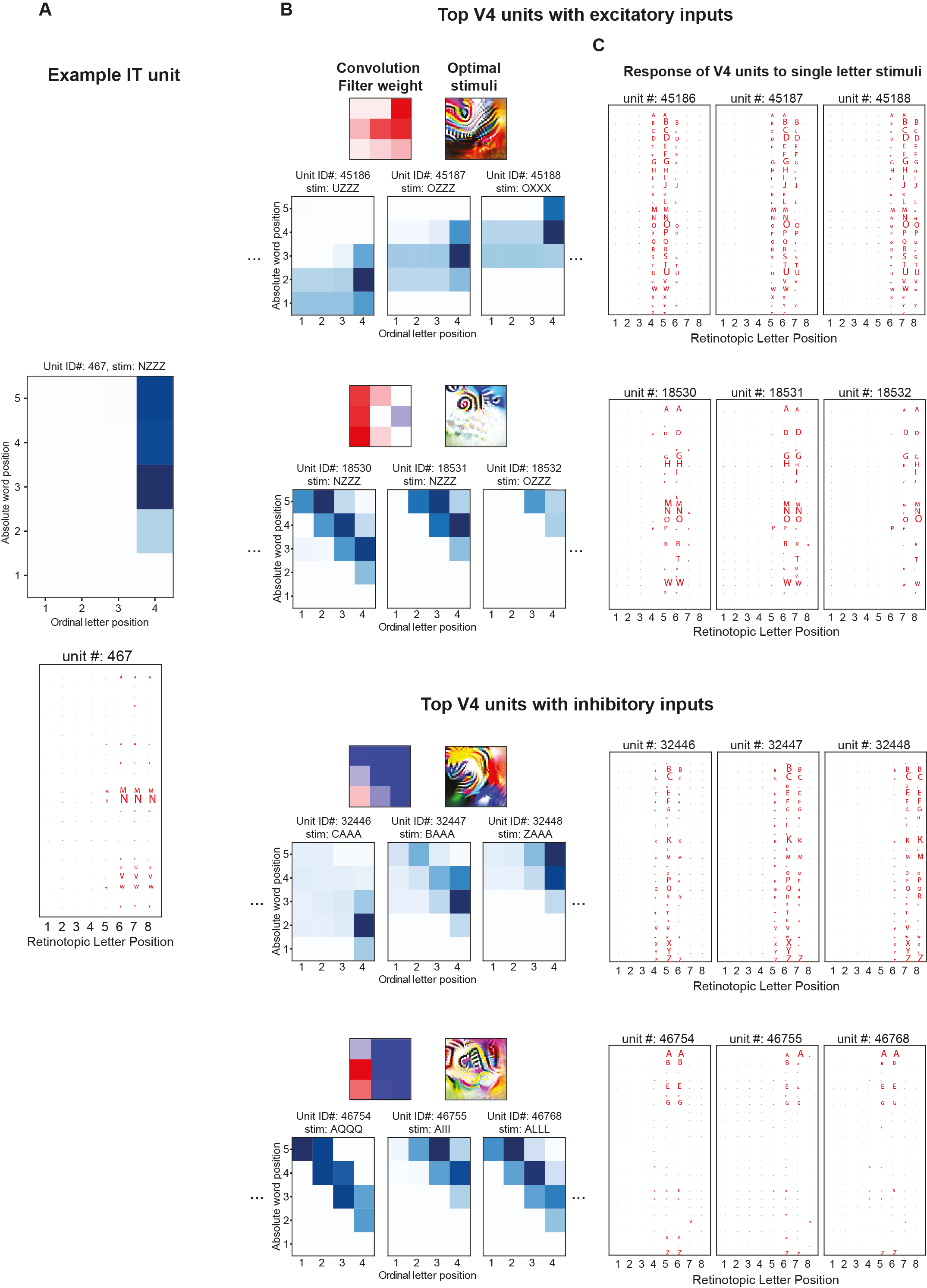


**Fig D:** **Functional connectivity between V4 and IT units (edge position tuning).**

1. Response profile of an example IT unit that encodes ordinal position. The unit is most responsive to letter N in the last position. Normalized response profile to single letters stimuli presented at different spatial locations suggest that this unit is weakly selective to other letters containing ‘V’ shaped structure.
2. Response profile of word-selective units in the V4 layer to their preferred stimuli that lie within the receptive field of the specific IT unit. Two V4 channels with excitatory inputs (top) and inhibitory inputs (bottom) to the IT unit are shown. For each channel, we visualized the convolution filter weights that connect V4 and IT layer and generated the stimuli that would maximally activate a given filter using activation-maximization method. Additionally, we visualized the response profile of three sample units. For example, the 3 units on the top row are sensitive to several angular letters including U and O (see panel C), but only if they appear left of a space. The 3 units on the second row are highly sensitive to letter N regardless of where it appears.
3. Normalized response profile of the units shown in (B) to single-letter stimuli presented independently at different spatial locations.


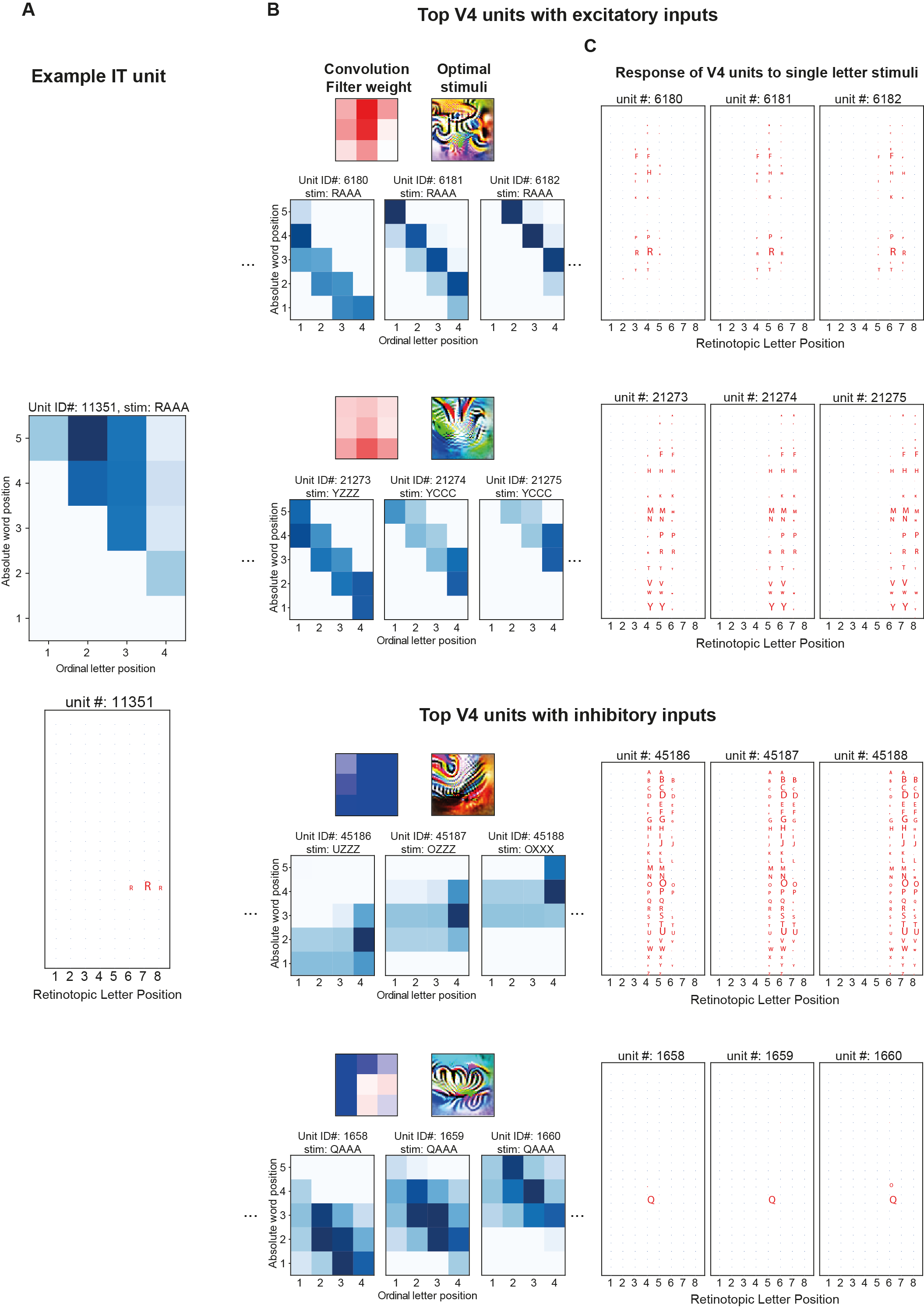


**Fig E: Functional connectivity between V4 and IT units (mid-position tuning).** Similar to Fig D, but depicting an IT unit selective for a preferred letter in middle positions. This unit receives excitatory inputs from retinotopic V4 units and inhibitory inputs from V4 units encoding space bigrams.

**Section B: Connectivity between V4 and V2 units**


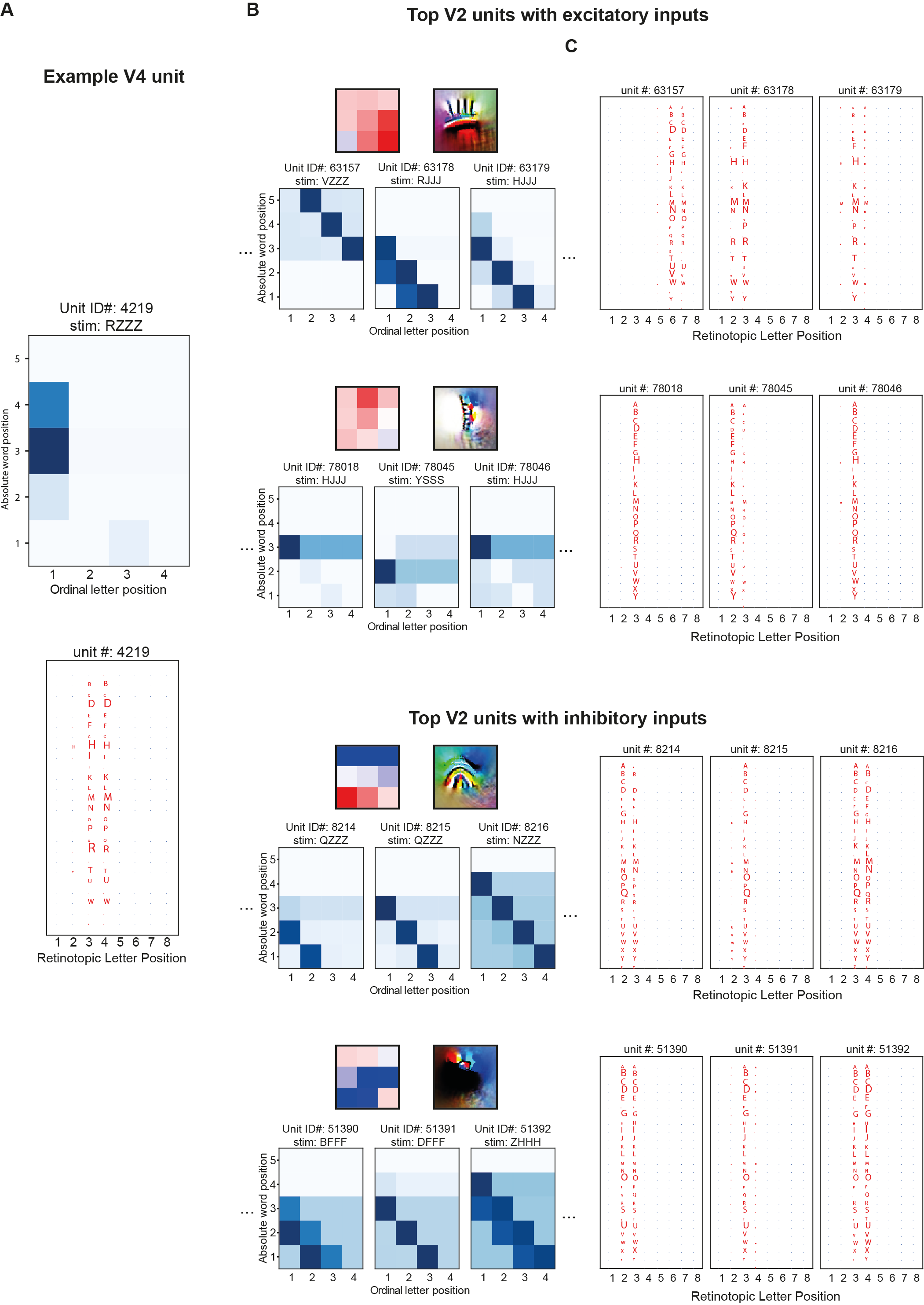


**Fig F: Functional connectivity between V2 and V4 units (edge position tuning).** Same as Fig D but for a V4 unit encoding edge position.


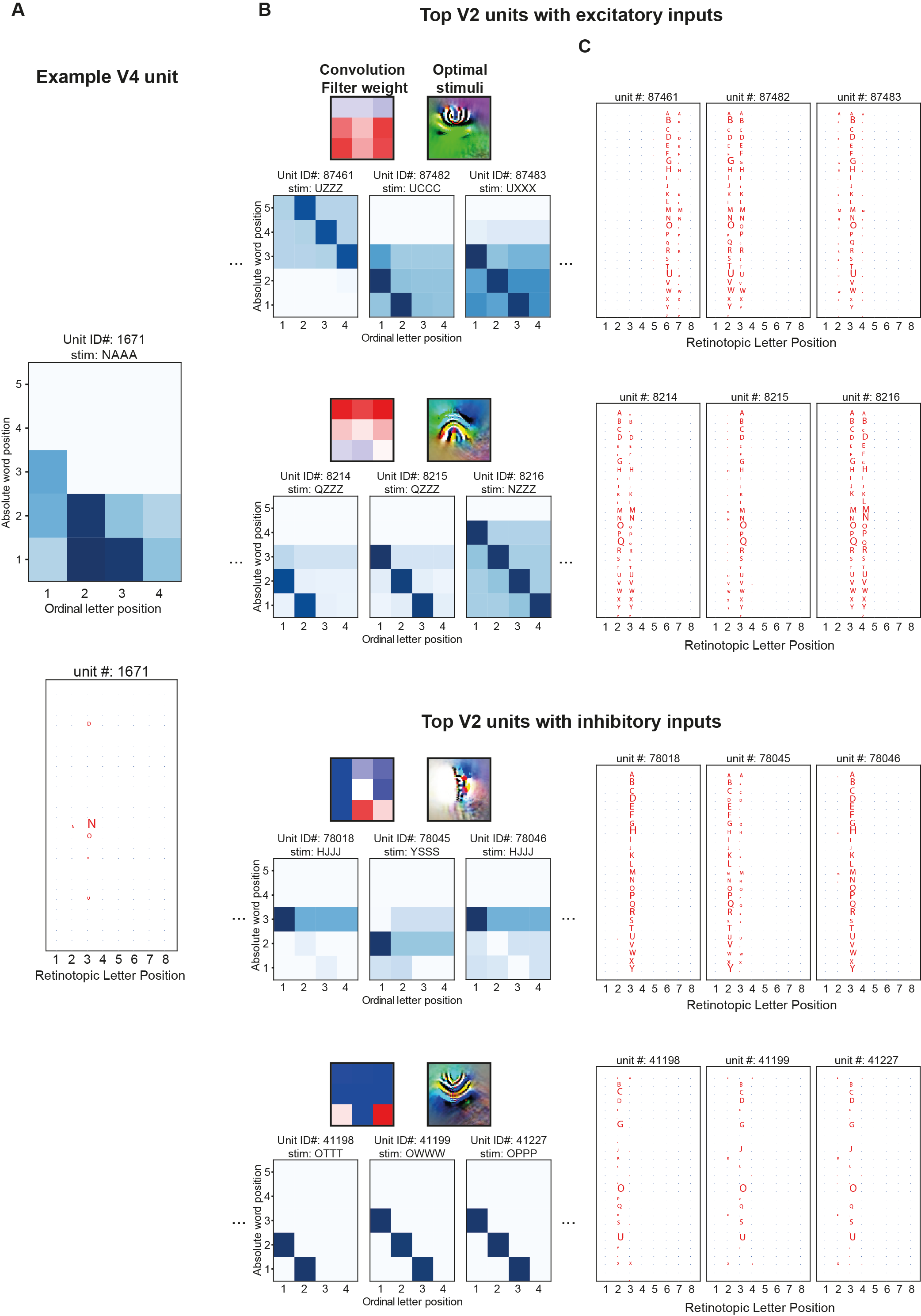
 **Fig G: Functional connectivity between V2 and V4 units (mid-position tuning).** Same as Fig E but for a V4 unit encoding mid positions.

**Section C: Emergence of Blank Space Coding Units**

Having established the link between space coding and ordinal position units in the higher layers of the literate CNN, we next investigated the type of inputs from the V1 layer that leads to the formation of space coding units in the V2 layer. Since the V1 layer has very few word-selective units, we could not perform the same analysis as just described. Instead, we initially visualized the weights of the V1 layer to gain insights into which features in the image would elicit the maximum response (Fig C). To ensure all filter weights fell within the range [0, 255], any negative values were adjusted by adding the minimum value and normalizing the maximum value. This analysis revealed a classical array of early visual features, including Gabor-like filters and color boundaries.


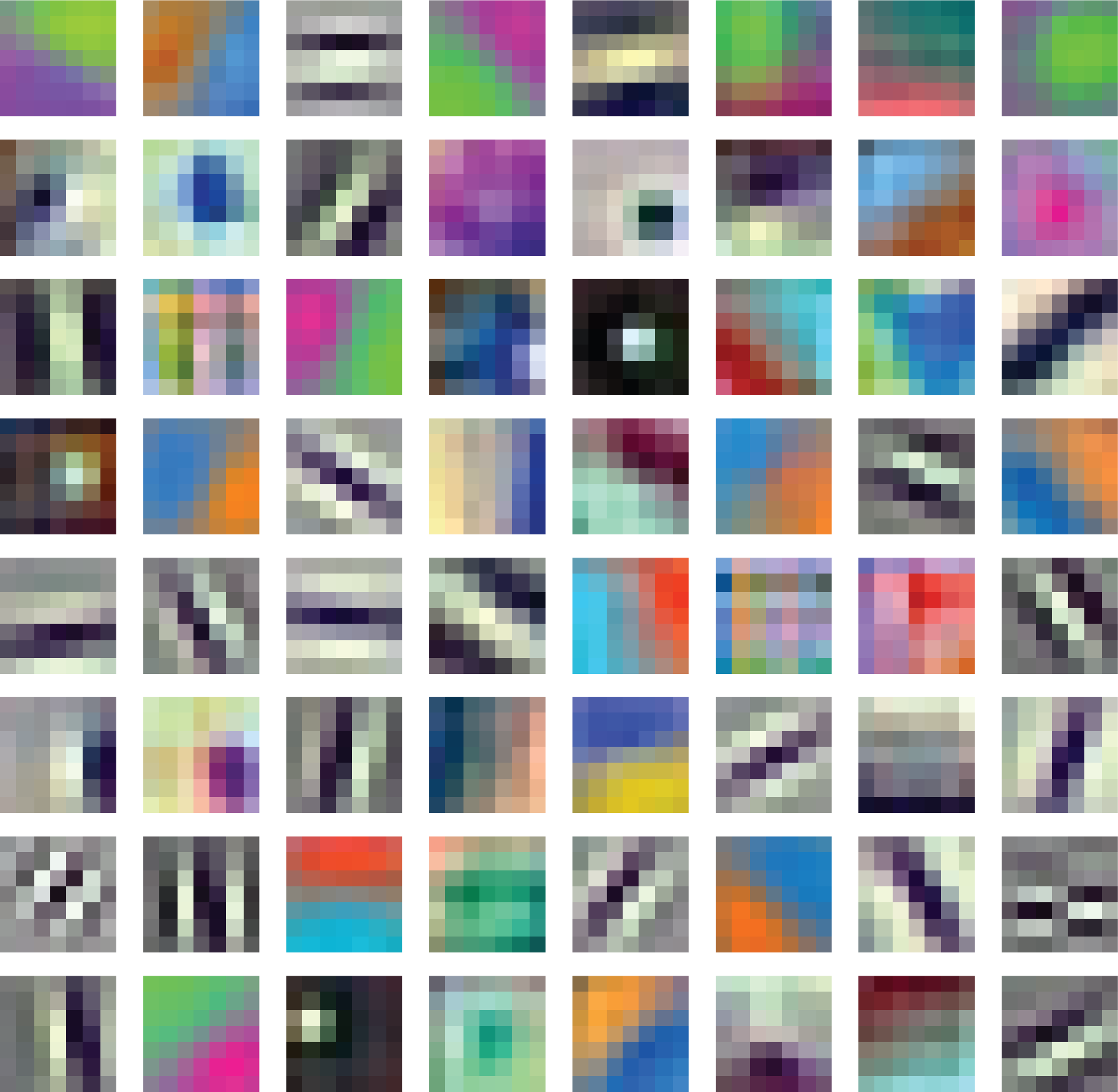


**Fig H: Visualization of V1 filters.** The V1 units comprise 64 distinct filters, each of size 7x7, whose weights are modified during training. Each filter represents the type of input image that would evoke a maximum response.

Following this, for any given V2 unit, as in our earlier approach, we summed the weights of its convolution filter from V1, and estimated the input activation vector across all units within its receptive field. For each V2 unit, we identified the top-5 inputs from V1 that resulted from the product of summed convolutional filter weights and V1 unit activations (see S4 Fig for 4 example units). Interestingly, we observed that the retinotopic units in V2 (bottom two example units in Fig D) are primarily driven by V1 units equipped with ridge detector filters. Such V1 units are ideally suited to detect individual character parts, since they respond to high-spatial-frequency features such as oriented bars or curves, whenever there is an intensity change from white to black to white again in a close sequence. Such features are characteristic of the fine strokes and curves that make up characters in text. By contrast, we found that V1 units predominantly influence space coding units (top two example units in Fig D) with edge-detection filters, i.e. low-spatial-frequency filters that encode the broader transitions between white and black pixels. Such filters are most suited to detect the overall shape or contour of a word and especially the location of its beginning and ending.


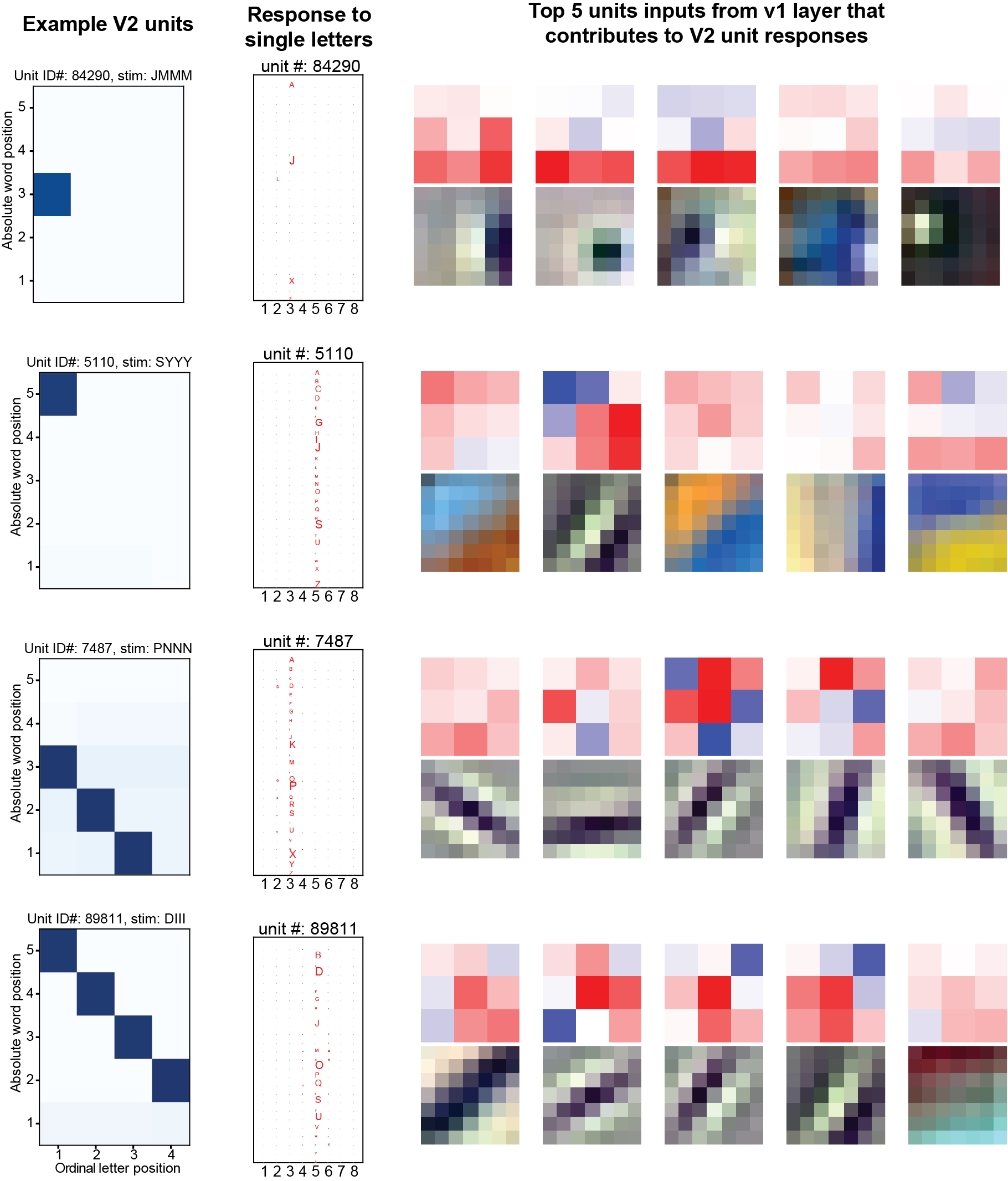


**Fig I: Functional connectivity between V1 and V2 layers.** The figure shows the responses of four V2 units: two space coding units (top rows) and two retinotopic coding units (bottom rows). The units are characterized by their responses to 4-letter stimuli dissociating word position and ordinal letter position (left column; see Fig 3A) and to individual letters at each of 8 retinotopic positions (second column). The right-hand side shows the top-5 convolutional filter weights between V1-V2 and corresponding features in the image that would elicit maximum response.


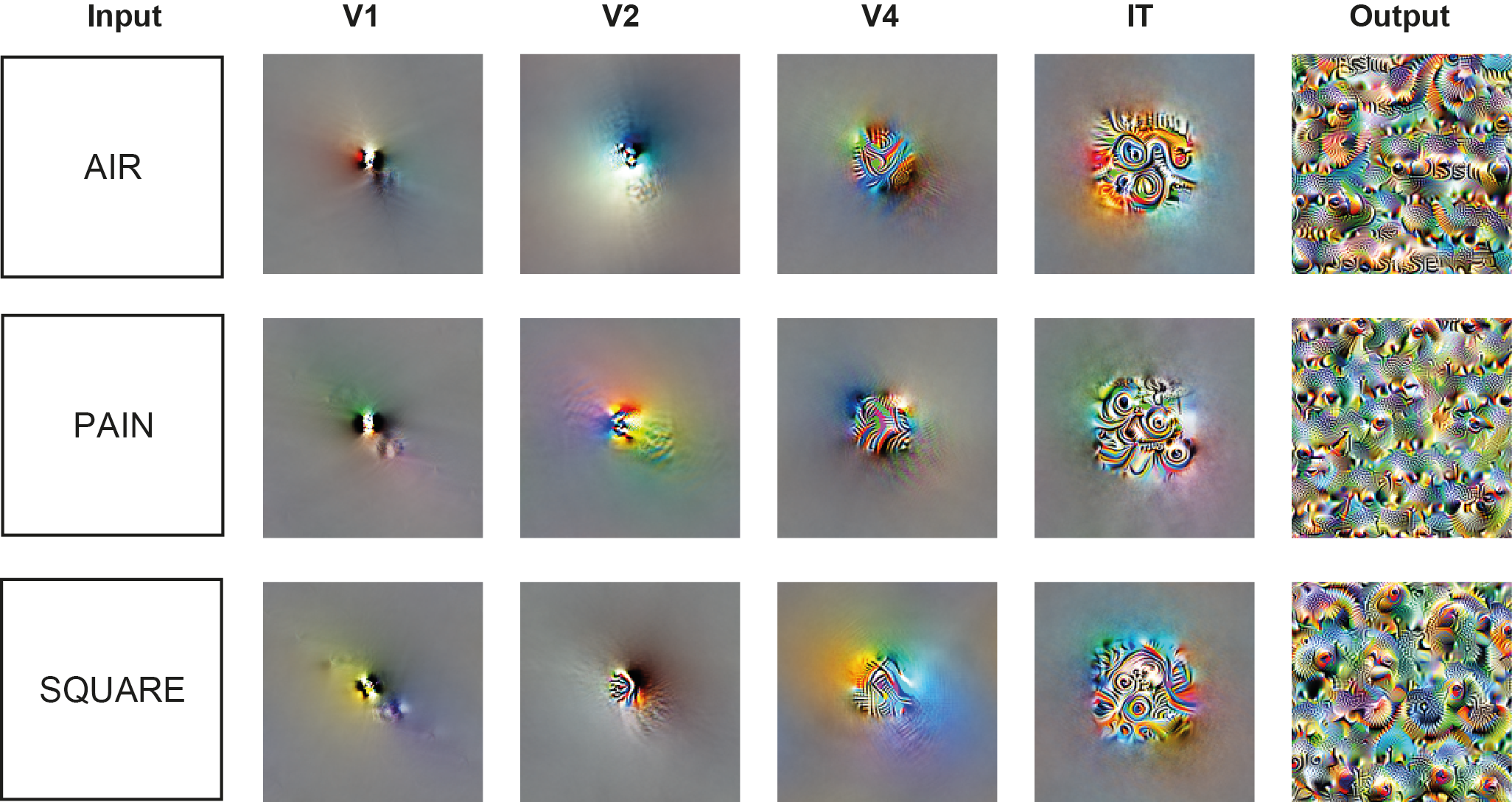


**Fig J: Activation-maximization of word selective units (negative direction).** Same as Fig 6, but visualizing the negative direction (or inhibitory stimuli) of each channel that evoked the highest response within a given layer.
