# Supplementary material for: Cracking the neural code for word recognition in convolutional neural networks
Source: PLoS Comput Biol. 2024 Sep 6;20(9):e1012430. doi: 10.1371/journal.pcbi.1012430 (PMC11410253; doi:10.1371/journal.pcbi.1012430)
Supplement: Attachment — Submitted filename: plos.computational.biology.pdf [file pcbi.1012430.s002.pdf]

The authors have carried out a systematic analysis of how a CNN (one claimed to mirror human vision) encodes letter identity and letter positions and develop a novel theoretical account of how words are encoded orthographically. I enjoyed the paper and think it will make an excellent contribution to PLOS Computational Biology. There are few points I would like clarified and some issues that I think are somewhat problematic for their account. But even if not resolved, I would still recommend publishing as long as the potential limitations are discussed (or addressed).

The critical problem I see is that the evidence that letter transpositions effects are larger in medial compared to external positions is not so clear-cut. Indeed, in the Adelman et al. (2014) mega-dataset of form priming effects, similar TL priming effects were observed across positions, with the largest effect (by small margin) in the final position. This is at odds with the authors analyses of the learned representations and the theoretical account they develop. In addition, in the Yin et al. (2023) paper the authors cite, similar TL priming effects were obtained across positions in a range of CNNs. Again, this seems problematic for the authors analysis. Some discussion of this is merited.

Related to this, the authors predict that their analysis will extend to other writing systems. One important finding is that TL effects are much reduced in Hebrew and other writing systems in which transpositions often result in other words. It is hard to see how the space representations used to develop ordinal letter codes can lead to highly position specific letter codes for internal letters in these orthographies. Perhaps the model can account for this, but it would be interesting to discuss this contrast between orthographic systems and how the current analysis can account for the effect, or alternatively, how this finding poses a challenge for it.

The authors write: “Thus, the ordinal positions of all letters in a word can be extracted in a fast, parallel, feedforward manner”. Could the authors elaborate a bit more about how the ordinal position of letters are encoded for the medial letters?

The following is less important, but perhaps interesting. The authors report figures that show that there are highly specific letter units (independent of position) as well as position specific units (that encode different letters). This contrasts with the failure to find “grandmother” cells in CNNs trained to classify objects, as reported by Gale et al. (2020). One thing to note is that we reported that different measures provided quite different estimates of selectivity – on some measures some units were highly selective and on other measures they were not. And in fact, when we simply plotted the activation of units in response to all images it is clear that no unit selectively coded one category of object. It would be interesting to know if the letter units are more selective than the object representations that we recorded from, and the way to assess this is to simply plot the activation of a few units in the way we did, as depicted in figure below (or use other numerical measures of selectivity that we report).

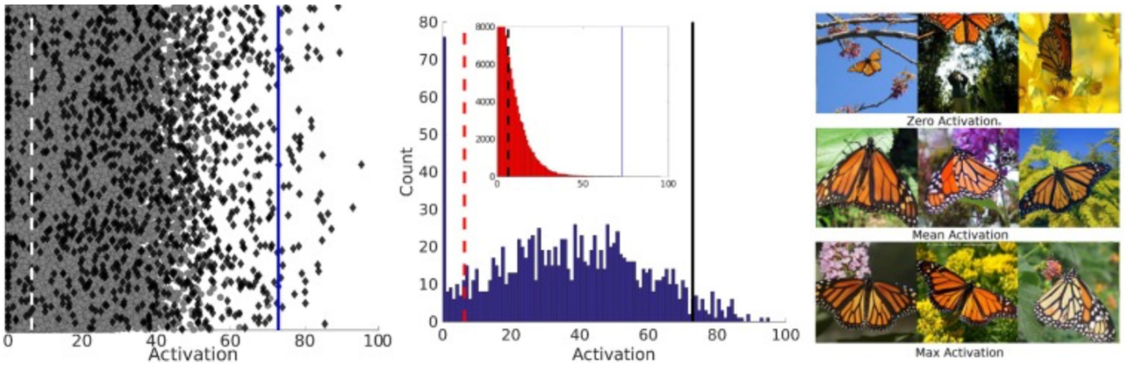

Download : Download high-res image (573KB)
Download : Download full-size image

Fig. 4. Data for unit $fc6_{1199}$. **Left:** activation jitterplot, black diamonds: Monarch butterfly images; grey circles: all other classes; white dashed line: threshold for the butterfly class maximum informedness; blue solid line: threshold for top 60 activations. **Middle:** histogram of activations of Monarch butterflies; red dashed line: threshold for the butterfly class maximum informedness; black solid line: threshold for top 60 activations. **Inset:** zoomed-in histogram of all activations across all ImageNet classes of unit $fc6_{1199}$ (N.B. this plot shows only the highest 121,586 activations; there are 596,734 activations at 0). There are Monarch butterfly images covering the whole range of values, with 72 images (5.8% of the total) having an activation of 0. **Right:** example ImageNet images with activations of 0 (top), the mean, 39.2±0.6, (middle), and the maximum, 95, (bottom) of the range. Although the high precision score suggests that this unit is a butterfly detector this is misleading given there are butterfly images over the entire activation range (including 0).

Line 273, typo: "by pulling over several blank-space units from the preceding layers" – should be pooling

Signed: Jeffrey Bowers

References:

Adelman et al. (2014) A behavioral database for masked form priming. Behavior Research Methods, 46(4):1052–1067, 2014. doi: 10.3758/s13428-013-0442-y.

Gale, E. M., Martin, N., Blything, R., Nguyen, A., & Bowers, J. S. (2020). Are there any 'object detectors' in the hidden layers of CNNs trained to identify objects or scenes?. *Vision Research*, *176*, 60-71.
