# Supplementary material for: Cracking the neural code for word recognition in convolutional neural networks
Source: PLoS Comput Biol. 2024 Sep 6;20(9):e1012430. doi: 10.1371/journal.pcbi.1012430 (PMC11410253; doi:10.1371/journal.pcbi.1012430)
Supplement: Attachment — Submitted filename: reviewer_response.pdf [file pcbi.1012430.s004.pdf]

We thank both the reviewers for their valuable comments. We have tried our best to incorporate all the suggestions and it has significantly strengthened the overall conclusion of our study. Below are the detailed responses to the questions raised by the reviewers.

Reviewer 1:

The authors have carried out a systematic analysis of how a CNN (one claimed to mirror human vision) encodes letter identity and letter positions and develop a novel theoretical account of how words are encoded orthographically. I enjoyed the paper and think it will make an excellent contribution to PLOS Computational Biology. There are few points I would like clarified and some issues that I think are somewhat problematic for their account. But even if not resolved, I would still recommend publishing as long as the potential limitations are discussed (or addressed).

The critical problem I see is that the evidence that letter transpositions effects are larger in medial compared to external positions is not so clear-cut. Indeed, in the Adelman et al. (2014) mega-dataset of form priming effects, similar TL priming effects were observed across positions, with the largest effect (by small margin) in the final position. This is at odds with the authors analyses of the learned representations and the theoretical account they develop. In addition, in the Yin et al. (2023) paper the authors cite, similar TL priming effects were obtained across positions in a range of CNNs. Again, this seems problematic for the authors analysis. Some discussion of this is merited.

Although the effect size may be small, the evidence for stronger priming from medial letter transposition compared to initial letter transposition is consistent across several studies. Even in Adelman (2014) (or Table 1 of Yin et al. (2023)), the Priming score for initial transposition (29.0) is ~8% lower than the Medial transposition (31.4). It is surprising that the Final transposition has a stronger priming score (32.5). This is inconsistent with Xiong et al., who found the same pattern of reading cost (initial > final > internal) for central and peripheral vision. The magnitude of effect size could vary with the task demands and is now included in the discussion.

Related to this, the authors predict that their analysis will extend to other writing systems. One important finding is that TL effects are much reduced in Hebrew and other writing systems in which transpositions often result in other words. It is hard to see how the space representations used to develop ordinal letter codes can lead to highly position specific letter codes for internal letters in these orthographies. Perhaps the model can account for this, but it would be interesting to discuss this contrast between orthographic systems and how the current analysis can account for the effect, or alternatively, how this finding poses a challenge for it.

The reduced transposed letter (TL) effects in Semitic languages (like Hebrew) are specific to lexical decision tasks and can be restored using a same-different task (Kinoshita et al., 2012). Thus, the generalizability of the proposed model to different languages is restricted to the mechanisms associated with bottom-up processing independent of any lexical access. Further studies should investigate the changes in representation space across different task conditions. We have elaborated the discussion section to include these points.

The authors write: “Thus, the ordinal positions of all letters in a word can be extracted in a fast, parallel, feedforward manner”. Could the authors elaborate a bit more about how the ordinal position of letters are encoded for the medial letters?

In the supplementary Figure 1, we report other kinds of units with mixed selectivity that encode medial position with less precision (Figure 2C). To investigate the emergence of such units, we performed additional analysis (Figure S5) and found that these units receive inhibitory inputs from edge coding units thereby enhancing their tuning for medial positions. Thus, edge coding units act as anchors, and the medial positions are encoded relative to them. The result section is now updated to include these findings

The following is less important, but perhaps interesting. The authors report figures that show that there are highly specific letter units (independent of position) as well as position specific units (that encode different letters). This contrasts with the failure to find "grandmother" cells in CNNs trained to classify objects, as reported by Gale et al. (2020). One thing to note is that we reported that different measures provided quite different estimates of selectivity – on some measures some units were highly selective and on other measures they were not. And in fact, when we simply plotted the activation of units in response to all images it is clear that no unit selectively coded one category of object. It would be interesting to know if the letter units are more selective than the object representations that we recorded from, and the way to assess this is to simply plot the activation of a few units in the way we did, as depicted in figure below (or use other numerical measures of selectivity that we report).

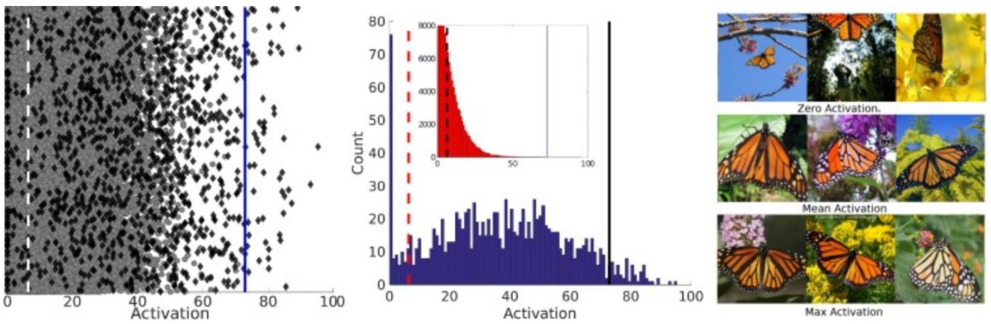

Download : Download high-res image (573KB)

Download : Download full-size image

Fig. 4. Data for unit $fc6_{1199}$. **Left:** activation jitterplot, black diamonds: Monarch butterfly images; grey circles: all other classes; white dashed line: threshold for the butterfly class maximum informedness; blue solid line: threshold for top 60 activations. **Middle:** histogram of activations of Monarch butterflies; red dashed line: threshold for the butterfly class maximum informedness; black solid line: threshold for top 60 activations. **Inset:** zoomed-in histogram of all activations across all ImageNet classes of unit $fc6_{1199}$ (N.B. this plot shows only the highest 121,586 activations; there are 596,734 activations at 0). There are Monarch butterfly images covering the whole range of values, with 72 images (5.8% of the total) having an activation of 0. **Right:** example ImageNet images with activations of 0 (top), the mean, 39.2±0.6, (middle), and the maximum, 95, (bottom) of the range. Although the high precision score suggests that this unit is a butterfly detector this is misleading given there are butterfly images over the entire activation range (including 0).

We would like to emphasize that the evidence for letter-selective units is not equivalent to grandmother cells. In our training dataset, the analogous of butterfly would be a whole word and we do not report evidence of units that specifically encode one particular word category. Further, units that like one particular letter (say O) would likely respond to a wide range of objects that contain similar features (e.g. Ball for units encoding O). We have elaborated the discussion section to include these points.

Line 273, typo: "by pulling over several blank-space units from the preceding layers" – should be pooling
Yes. We have corrected it now.

Signed: Jeffrey Bowers

References:

Adelman et al. (2014) A behavioral database for masked form priming. Behavior Research Methods, 46(4):1052–1067, 2014. doi: 10.3758/s13428-013-0442-y.

Gale, E. M., Martin, N., Blything, R., Nguyen, A., & Bowers, J. S. (2020). Are there any 'object detectors' in the hidden layers of CNNs trained to identify objects or scenes?. *Vision Research*, *176*, 60-71.

Reviewer 2

Reviewer's summary

This work approaches the question how an invariant ordinal code for letter strings is achieved by neural circuits using convolutional neural networks. The authors investigate the unit's properties of several literate networks and discuss how they align with human word recognition. Furthermore, a new coding principle is discovered, that integrates 'spaces' in the positional encoding of letters. The results tie together into a final understanding of how low-level information is combined by learnt features to achieve invariant word recognition. The novelty of this work is the use of CNNs to investigate the emergence of codes for written words without hand-crafting features (as was common in cognitive models).

Introduction

1 L71-74 "*Here, we show that precise predictions about the neurophysiological architecture for invariant reading can be obtained by studying convolutional neural networks (CNN) models of the ventral visual cortex that, like literate humans, can be recycled for reading*."

If the term 'recycling' in this context refers to 'neural recycling, it would be good to rephrase the sentence such that the term is applied to neural representations. Now it ambiguously implies that entire networks or literate humans are recycled.

This sentence is now rephrased as "Here, we show that precise predictions about the neurophysiological architecture for invariant reading can be obtained by studying convolutional neural networks (CNN) models of the ventral visual cortex. Similar to literate humans, the neural representations of these networks can be recycled for reading."

Results

Invariant word identification.

2.1 When reading, I noticed I really required more information on the datasets and training to understand what you did in this section. The methods section provides sufficient details, but I would try to be a bit more explicit here in the results section. For example, it is not clear from the description in L83 whether you keep training on the image data once the 1000 words are introduced, while this is an important detail.

In addition, it is not directly clear what kind of image categories you are training on, as technically, the images of words are also image categories. This could be easily resolved by mentioning the dataset earlier.

Lastly, it seems from the text like you train several network instances on all languages at once (inspecting figure 1 and the Methods clarify that that is not the case). Perhaps you could differentiate between mono- and bilingual networks? In any case, I would recommend clarifying which networks you train on which data more clearly in the text.

The text is now updated to clarify these details

2.2 From (the caption of) figure 1A, it is not clear that the literate network is trained in two phases.

The main text now explicitly mentions this point

2.3 How are the lengths of the two training phases determined?

The length of the training phase was chosen to ensure that the accuracy reached its peak value and didn't improve any further. This was based on our earlier work (Hannagan et al. 2021) and is clarified in the methods section.

Emergence of script-specific units.

3.1 L103-104. "*Although few, there were units in each network that exhibited selectivity to novel scripts but not the trained script*" What does the selectivity tuning of these units look like? I see you describe how similarity between scripts influences these responses. It would be interesting to know if scripts are more likely to activate the same subnetworks and features in earlier layers of the CNN if scripts are more similar with regards to their low-level features. Perhaps you can these results together with your activation maximization analysis, in which you only consider the monolingual French network?

Investigating the tuning of units selective to a novel script (non-Latin in the case of the French network) is a complex task. It requires fitting linear models for words from those scripts and visualizing the model parameters, which is challenging due to the non-linear nature of the Indian writing system, where diacritics can appear on all four sides of the base letter. This complexity necessitates extensive investigation to build accurate models. While this is an important direction for future research, it is beyond the scope of the current study. Additionally, investigating other networks would lead to more questions than we can address within this work.

3.2 L113-114. Is it possible to distinguish two languages that use the same script in the network representations (e.g., French and English) or does the network treat them as one?

When two languages have the same script, they rely on the same set of units that encode letter identity and position. This is evidenced by ~100% overlap in the word-selective units for French and English. However, these networks also learn the statistics of words within a language

(Hannagan et al. 2021). Thus, if we chose a large set of words with varying statistics, it should be possible to obtain above chance performance in classifying two distinct languages. We indeed observed above-chance classification accuracy (especially in later layers of the French network) (Figure S1). The results section is now updated to include this finding.

3.3 Figure 1B. How would you explain the difference in English-selective units between English+Chinese and English+French? In this plot it is unclear how many units are selective to multiple languages, so a unit can be counted multiple times, is that correct? i.e. when inspecting the total number of units selective to French AND English, would they count up to the same number for the overlapping units in the English + Chinese and the English + French model? Relatedly, I observe that the number of French and English units is always roughly the same in each network, do you know why?

Yes, the plot only indicates the number of units that are selective to a given language, and the same unit is often counted multiple times. The difference in English selective units could be a consequence of a higher number of Chinese selective units and since English script shares some features with Chinese, it resulted in a higher number of English selective units as well. We have elaborated the results section to address this in detail.

Improvements in neural discriminability.

4.1 L134. As you mention, perceived dissimilarity between letters, and not only bigrams and words, increases with literacy. It would thus be interesting in seeing if this effect applies to your networks as well. This could give more insight into the granularity at which it is learning orthographic features.

We have now included this analysis as a part of the supplementary section. The results were consistent with our findings using bigram stimuli.

4.2.1 Related: It is not entirely clear why you conduct the bigram comparison in the French network only (versus illiterate network), but the word comparison between two literate networks. To match your claims about similarity to human neural responses, this could benefit from better control analyses.

Sorry for the confusion. We indeed compared bigrams representation between two literate networks as well. This was mentioned in the figure caption and is now corrected under the results sections.

4.2.2 The caption of 1D says the dissimilarity is calculated between bigram pairs, while the text (L139-140) seems to indicate the analysis is between words?

This is a typo; we used the same stimuli as tested in the human experiments.

4.3 L145. How many unique symbols do the other languages have, and how does it impact your analyses?

The number of unique symbols would depend on the approach towards counting them. For e.g., Telugu script has approximately 60 primary symbols, but when considering all the combinations with vowel signs and other diacritical marks, the number of unique syllables can exceed 500. Same is true for Malayalam as well. The impact of the number of symbols on neural discriminability is unclear and beyond the scope of this study. This is now explicitly mentioned in the main text.

Letter tuning.

5.1 L154-158. Human readers show sensitivity to edge letters. It is interesting that you find such sharp position tuning and sensitivity to the beginning and ending of sentences in a feedforward network too. In humans, this effect is also connected to the linear and temporal aspect of reading, which the CNN does not have. Do you think it has to do with higher contrast at the edges / "crowding" in the centre?

Yes, we speculate that the presence of blank space coding units (higher contrast at the edges) might play a key role in this edge selectivity. We had mentioned this in the discussion section "This code is extracted by a feedforward hierarchy of neurons with increasingly larger retinotopic receptive fields, jointly sensitive to the high-frequency shapes that make up letters and to the low-frequency patterns that signal word boundaries (Figure 7) (see Schubert et al., 2021 for a similar finding)."

5.2 Figure 2A. It is rarely mentioned how/in what unit you measure selectivity, that would be helpful to mention in the caption for this figure specifically.

The figure caption is updated to clarify it.

5.3 Figure 2C is a bit confusing and crowded (although the caption and methods clarify well). I recommend giving it a more informative title than 'mean normalized coefficient' (and put that as axis label instead).

Figure 2C is now updated

Emergence of letter- and position-invariant units.

6.1 Figure 3:

6.1.1 A small detail that does not require change: flipping the axes of these matrices would be more intuitive to me (absolute word position on the x-axis, letter ordinal position as y-axis.

Thank you for the suggestion.

6.1.2 Axis labels would be helpful in 3B, 3C, and 3D too.

Figure 3 is now updated.

6.1.3 For clarification on figure 3B: you show 2 *types* of units, but 4 units (hence 4 colours)?

Yes, that is correct

6.2 It is great to see a few example units, but it would be helpful to get a global measure to inspect the progression from early to later layers, (which you mention in the text as percentages). Relatedly, is there a way to quantify the transition from retinotopic to ordinal, or do you have to handpick these units?

As you rightly mentioned, we have quantified the global progression from retinotopic to ordinal position coding, as mentioned in the text. To clarify, we did not handpick units for this analysis. Instead, we used an image processing-based approach to categorize each unit's response pattern as row, column, diagonal, or mixed.

Specifically, we analyzed each 5x4 response matrix (e.g., Figures 3C and 3D) as follows:

1. We transformed the matrix to binary elements. The threshold was set at 30% of the difference between the maximum and minimum values of that matrix.
2. Units with a maximum response of less than 5 were excluded from the analysis.
3. We developed custom scripts to categorize each binary matrix as word position selective (row structure), ordinal letter position (column structure), retinotopic letter position (diagonal structure), or mixed selectivity (mixed structure).

This systematic approach allowed us to quantify the transition from retinotopic to ordinal coding across layers without any subjective bias. The methods section is now updated to clarify these steps.

Emergence of ordinal position coding units

7.1 Very nice analysis. It would be helpful to mention here explicitly that you choose V4 and IT because that is where you observe the transition from retinotopic to ordinal codes.

The text is now updated to state this explicitly.

Optimal stimuli for each layer

8.1 You wrote that all sections after Figure 1 would be performed on the French network, but you are using English word stimuli in Figure 6. Are you using the English network here instead?

No, these words are present in French as well and were part of the training set. These words overlap with English but some of them have a different meaning e.g., PAIN in French means bread. We have now clarified the figure caption.

8.2 L323-326 *"Remarkably, from IT and output, the automated image optimization process recovered word fragments that partially matched the word identity originally used to select the units"* This is interesting, and I am curious why. Is the repetition of the word in the visualisation explained by weight sharing upstream?

Yes, it is most likely the consequence of weight sharing and invariant representation in the later layers of the network. Such repetition has been observed in earlier research investigating object properties (https://distill.pub/2017/feature-visualization/). We have updated the text to elaborate on this.

8.3 The methods of visualization and unit selection are not entirely clear to me, and I have several questions about them. In the caption of Figure 6 you write: "*[..] the features of the channel whose units evoked the highest response within a given layer are shown*. *For visualization purposes, features are displayed at the central location*" And in Methods: "*Given the convolutional structure of the early stages of the network, all units within a given channel have the same features but at different spatial locations. Thus, we only estimated the preferred input for the unit whose receptive field was at the center of the image.*"

8.3.1 First, you mention the early stages of the network, but it's only the H layer and readout that are not convolutional, is that correct?

Yes, that is correct. We have rephrased the sentence to make it clear.

8.3.2 Secondly, "displaying the features in the centre" sounds like you are moving the visualization. If you are performing this analysis on a centre unit, which has a central RF, why do you need to move the visualisation?

We are indirectly moving the visualization. As stated in the methods, our goal is to "generate images that maximally activate a given unit". Within each layer, we select this unit based on the peak activity across all units. However, this unit's RF may not always map the center region. Thus, we optimize a neighboring unit whose receptive field is at the center. The text is now updated to explain this better.

8.3.3 The first sentence in the figure caption, which describes that you select the channel with the highest response in the layer, does not seem to match with the methods section where you write that you select the unit in the centre position for this analysis. Are you selecting the *n_channel* units that have the centre position (share RF) in the 3D layer, and from those, selecting the unit with the highest activation?

In this analysis, we are optimizing for the channel. For example, the output from the IT layer is a 512x7x7 tensor, where there are 512 channels, and each channel contains a 7x7 grid of

units that span the entire stimulus. First, we identify the unit with the highest activity, which can belong to any of the 512 channels and any of the 7x7 receptive field positions. For consistency, we then choose the center position within that channel for our visualization. The text is now updated to reflect this example.

8.3.4 In the methods you write: "*For ease of visualization, only the images generated along the positive directions are shown*." Do the images in the negative direction show anything interesting?

We have added a new figure indicating the negative direction in the supplementary section (Figure S10)

Methods

9.1 The first section writes: "IT, avgpool IT or H". I did not take away easily from your paper that H is the average pooling layer before the linear readout. It could be better described or visualised in architectural figure 1, that this is the pooling of IT with a new name. Additionally, since the other layers have names corresponding to visual cortex, it would be helpful to know the rationale behind naming this final layer 'H', and why you use it in your first analyses, and not IT. It would thus be great if you can include a clearer description of the layer choices, more details about the architecture (for example by adding a table with all layers and respective parameter details), and finally a justification of the use of the H layer in the first few analyses.

We have now updated the figure to indicate that the last layer is an average pooling layer. This architecture is part of the Cornet models (https://doi.org/10.1101/408385), which contains all the details about the architecture. However, the original cornet architecture has largely ignored the representation from AvgPool layer and didn't associate it with any region from the ventral visual pathway. We didn't have a strong rationale behind naming it 'H' and have now renamed it as avgIT (instead of H) throughout for a better naming convention.

9.2 With regards to the stimuli: the random flipping data augmentation was also not used training the illiterate network on Imagenet?

No. We have mentioned this in the methods section as well "For fair comparisons, other operations such as flipping were not performed on the Imagenet dataset as the same operation would create mirror words in the Word dataset, which is not typical in reading."

Minor details

10.1L167-177: "*each unit's receptive* [field]"

Updated
